# Molecular mechanisms underlying cashmere quality differences between Jiangnan cashmere goats and Changthangi pashmina goats

**DOI:** 10.3389/fvets.2025.1571803

**Published:** 2025-05-28

**Authors:** Gao Gong, Shijie Bi, Xin Liang, Yu Ao, Feng Xu, Yiming Sulaiman

**Affiliations:** ^1^College of Animal Science, Xinjiang Agricultural University, Urumqi, China; ^2^College of Food Science and Pharmacy, Xinjiang Agricultural University, Urumqi, China

**Keywords:** cashmere goats, skin transcriptome, PI3K-Akt signaling pathway, thermogenesis, KRTs

## Abstract

Cashmere goats are excellent livestock breeds known for producing high-quality cashmere fibers from secondary hair follicles. In this study, we aimed to explore the key RNA molecules responsible for the differences in cashmere quality between Jiangnan cashmere goats (JNCG) and Changthangi pashmina goats (CPG). Skin transcriptomic data from the anagen, catagen, and telogen stages of hair follicle growth were retrieved from the SRA database for both JNCG and CPG. Bioinformatics analyses were conducted to identify key molecular differences underlying the variation in cashmere fiber quality. The results showed that there were 4,942 differentially expressed genes (DEGs) between JNCG and CPG through differential analysis, and the DEGs were mainly enriched in PI3K-Akt signaling pathway, Thermogenesis, ECM-receptor interaction in KEGG through functional enrichment analysis, and GO entries were mainly enriched in keratin filament, intermediate filament, keratinization. Twenty-four key candidate genes including *IFG1*, *IGF1R*, *FGF5*, *FGF21*, *ND2*, *COX2*, *KRT10*, *KRT39*, and *KRT74* were further mined through pathways and entries. These genes play an important role in the development of secondary hair follicles and the formation of cashmere quality in cashmere goats, providing a theoretical basis for the genetic improvement of cashmere goats in the future.

## 1 Introduction

The cashmere goat is a breed primarily bred for cashmere production, while also being used for both meat and cashmere, making it an important component of the livestock industry. Cashmere goats are found in countries such as China, India, Iran, Pakistan, and Mongolia. The hair of cashmere goats is unique, characterized by a typical heterogeneous coat. Primary hair follicles (PHFs) produce coarse, medullated hair, while secondary hair follicles (SHFs) produce cashmere, which is fine and non-medullated. Cashmere is produced by the SHFs in the skin of cashmere goats, and the fibers are soft, fine, lustrous, warm, comfortable, lightweight, and elastic, making it the highest quality natural fiber material in the textile industry, often referred to as the “fiber gem” or “soft gold” (1–3).

Jiangnan cashmere goat (JNCG) is a newly developed breed, with the male parent being the Liaoning cashmere goat and the female parent being the Xinjiang goat, primarily aimed at increasing cashmere production while maintaining fiber fineness and ensuring post-harvest body weight. It has white-colored cashmere, high yield, stable genetic traits, and is adapted to the arid, semi-arid, and shrubland environments of extreme ecological regions in China, with a cashmere yield of 350–500 g and an average fiber diameter of approximately 15.2 μm (4, 5). The Changthangi pashmina goat (CPG) is a genetically significant breed, adapted to the harsh, cold, dry, and high-altitude climatic conditions of the Ladakh region in India, producing the world’s finest and most expensive animal fiber (pashmina), with an average fiber diameter ranging from 11–14 μm and a cashmere yield of 70–500 g (6, 7).

SHFs are unique, renewable organs. After birth, the SHFs of cashmere goats undergo cyclical growth phases of anagen, catagen, and telogen annually. The growth and development of these follicles are closely related to the length, fineness, and yield of cashmere fibers (8, 9). Numerous studies have shown that the cyclical growth and quality formation of SHFs in cashmere goats are regulated by various molecular factors, including hormonal regulation, such as melatonin (MT) (10), prolactin (PRL) (11), and growth factors, such as insulin-like growth factors (IGFs) (12) and fibroblast growth factors (FGFs) (13). In addition, keratin genes (KRTs) (14), keratin-associated proteins (KRTAPs) (15), and bone morphogenetic proteins (BMPs) (16) have been shown to regulate hair follicle cycles. The PI3K-Akt signaling pathway, MAPK signaling pathway, and cell cycle pathways also play important roles in hair follicle development (17, 18).

This study aims to select JNCG from China and the CPG from India as research subjects. Through the use of public databases, we analyzed the transcriptome data of skin tissues from both JNCG and CPG at different growth stages (anagen, catagen, and telogen). This bioinformatics analysis was used to identify transcriptomic differences between the two breeds and explore the key RNA molecules that contribute to the differences in cashmere quality. The findings will provide a theoretical foundation for improving cashmere fiber quality and breeding strategies for high-quality cashmere goats.

## 2 Materials and methods

### 2.1 RNA-Seq datasets

The RNA-Seq datasets was retrieved from the NCBI SRA database, specifically targeting skin RNA-Seq data for Chanthangi pashmina goat (CPG) and Jiangnan cashmere goat (JNCG). A total of three BioProjects were included: PRJNA688899, PRJNA480975, and PRJNA778726, comprising 44 samples in total. Among these, 30 samples were from CPG, including 16 from anagen, 2 from catagen, and 12 from telogen stages; while 14 samples were from JNCG, including 8 from anagen, 3 from catagen, and 3 from telogen stages (Table 1). All RNA-Seq data were generated using the Illumina platform with paired-end sequencing. Detailed data can be found in Supplementary Table 1.

**Table 1:** BioProjects detailed sample information table.

BioProject	Breed	Tissue	Number	Sample collection protocols and experimental designs
PRJNA688899	Chanthangi pashmina goat	Skin	19	Ten unrelated pashmina goats of the same age (24 months) were repeatedly sampled during anagen (October) and telogen (March). The skin samples containing pashmina follicles were collected from the flanking region of each goat
PRJNA480975	Chanthangi pashmina goat	Skin	11	Ten unrelated pashmina goats of the same age (26 months) were repeatedly sampled during early anagen, late anagen, catagen, and telogen. The skin sample was collected from the flanking region of each goat at same time point. Prior to sampling, the site was sheared, shaved and locally anesthetized with 2% lignocaine. Approximately 7 mm diameter skin samples were harvested aseptically with a single-use biopsy punch
PRJNA778726	Jiangnan cashmere goat	Skin	14	Ten unrelated Jiangnan cashmere goat of the same age (24 months) were repeatedly sampled during anagen, catagen, and telogen. Experimental cashmere goats with high production features were collected from breeding farm in breeding center of Wenshu County, Aksu Prefecture, Xinjiang Province. The cashmere goats were fed in accordance with the standards of Xinjiang. Three female adults were chosen (two-year-old Jiangnan cashmere goat with a coefficient of relationship of < 0.125)

### 2.2 Sequencing data quality assessment and quality control

To ensure the quality and reliability of the data analysis, raw data were evaluated and subjected to quality control to obtain clean reads. FastQC (v0.12.1) and Trimmomatic (v0.39) were used for quality control, with Trimmomatic’s key parameters set as: PE-phred33 ILLUMINACLIP:TruSeq3-PE.fa:2:30:10 LEADING:3 TRAILING:3 SLIDINGWINDOW:4:15 MINLEN:36.

### 2.3 Reference sequence alignment analysis

The reference genome and gene annotation files used in this study were from the goat reference genome (version: GCF_001704415.1_ARS1). Data analysis was performed using a high-performance computing node (Inspur, NF5280M6). The clean data for each sample were aligned to the reference genome using HISAT2 (v2.1.0) (19), with the following parameters: -p 5 --dta --phred33 --no-unal-t --un-conc-gz. BAM files were generated using Samtools (v1.6) (20), with the main parameter: samtools sort-O bam -@ 2-o. The BAM files were processed using StringTie (v2.2.1) (21), generating GTF files with the parameters: stringtie-e -B-p 5-G -o-A. A Python script was then used to process the StringTie output, calculate sample counts, and FPKM. Linux scripts and Python code are detailed in Supplementary Material 1.

### 2.4 Differential RNA expression analysis

The expression matrix of the samples was analyzed for differential expression significance. The analysis was conducted to identify differential genes between CPG and JNCG skin tissue samples at different stages: CPG_a vs. JNCG_a, CPG_c vs. JNCG_c, and CPG_t vs. JNCG_t. Differential expression analysis was performed using DESeq2 (v1.44.0) (22, 23) in R (v4.4.1), with the threshold for significant differential expression set at log_2_|(fold change)| >2 and adjusted *p*-value (*p*-adj) <0.01. Volcano plots of differential genes were generated using the ggplot2 (v3.5.1). Venn analysis was performed to obtain the union of the differential genes across all three groups, and a clustering heatmap of the differential genes was created using the pheatmap (v1.0.12).

### 2.5 Functional enrichment analysis

Functional enrichment analysis of all differential genes was performed using Gene Ontology (GO) and Kyoto Encyclopedia of Genes and Genomes (KEGG) pathway enrichment analyses. The DAVID (24) database was used for both GO and KEGG enrichment analysis, with a *p*-value <0.05 considered significant. A gene clustering heatmap of key pathways and functions was generated, and protein–protein interaction (PPI) network analysis was performed for these gene sets using the online STRING protein interaction database^1^ (25), with the species selected as *Capra hircus*.

## 3 Results

### 3.1 RNA-Seq data set analysis

**Figure 1:**
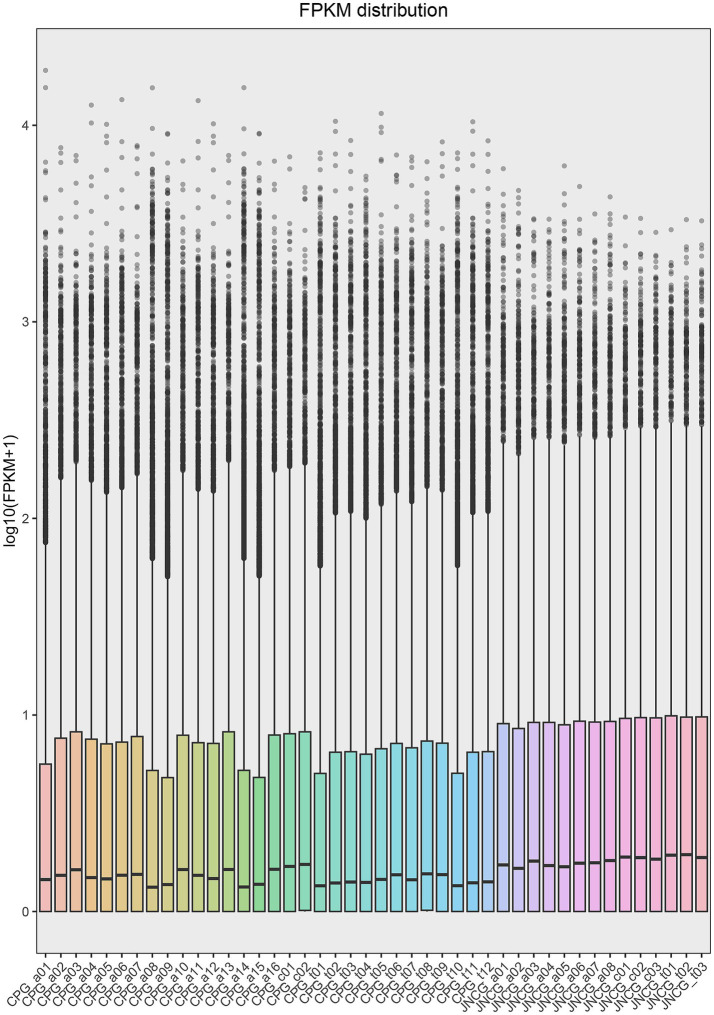
FPKM distribution map. The x-axis represents the samples, and the y-axis represents log_10_ (FPKM + 1).

In this study, RNA-Seq data from 44 samples were analyzed after quality control and filtering, a total of 1,928,029,516 reads were obtained. The clean data were mapped to the goat reference genome, with 1,881,690,918 reads successfully aligned, resulting in an overall alignment rate of 97.59%. The mapping rate for individual samples ranged from 93.46 to 98.22%, with an average mapping rate of 97.51% for CPG and 97.76% for JNCG (Table 2). The distribution of gene expression in each sample was analyzed using the FPKM values, and a violin plot of FPKM distribution was generated for all samples (Figure 1). The distribution of log_10_ (FPKM + 1) values was found to be consistent in size and distribution patterns across all groups.

**Table 2:** Samples mapped sequencing data with reference genome.

Sample	Total reads	Total mapped	Uniquely mapped	Multiple mapped
CPG_a01	42,182,888	39,424,127 (93.46%)	17,575,410 (41.66%)	20,752,952 (49.20%)
CPG_a02	45,457,857	44,262,315 (97.37%)	38,059,887 (83.73%)	4,693,107 (10.32%)
CPG_a03	45,420,019	44,334,480 (97.61%)	40,060,838 (88.20%)	2,865,952 (6.31%)
CPG_a04	46,977,577	45,774,951 (97.44%)	40,726,978 (86.69%)	3,656,702 (7.78%)
CPG_a05	40,881,717	39,929,172 (97.67%)	35,922,541 (87.87%)	2,787,607 (6.82%)
CPG_a06	31,241,865	29,979,693 (95.96%)	26,704,129 (85.48%)	2,395,323 (7.67%)
CPG_a07	49,137,005	47,903,666 (97.49%)	42,301,666 (86.09%)	4,141,889 (8.43%)
CPG_a08	53,095,764	51,768,369 (97.50%)	46,840,631 (88.22%)	3,801,254 (7.16%)
CPG_a09	33,080,790	32,339,780 (97.76%)	19,765,208 (59.75%)	11,615,549 (35.11%)
CPG_a10	40,937,293	40,147,203 (98.07%)	35,483,818 (86.68%)	3,689,310 (9.01%)
CPG_a11	19,401,828	18,901,260 (97.42%)	16,741,624 (86.29%)	1,497,263 (7.72%)
CPG_a12	41,333,783	40,395,506 (97.73%)	36,054,319 (87.23%)	2,806,282 (6.79%)
CPG_a13	35,369,862	34,559,892 (97.71%)	30,929,792 (87.45%)	2,211,438 (6.25%)
CPG_a14	52,719,209	51,438,132 (97.57%)	46,501,742 (88.21%)	3,781,601 (7.17%)
CPG_a15	33,222,925	32,515,276 (97.87%)	19,695,070 (59.28%)	11,625,132 (34.99%)
CPG_a16	41,227,654	40,464,942 (98.15%)	35,538,962 (86.20%)	3,709,056 (9.00%)
CPG_c01	48,291,329	47,407,597 (98.17%)	42,565,497 (88.14%)	3,497,105 (7.24%)
CPG_c02	60,994,927	59,860,421 (98.14%)	52,372,060 (85.86%)	5,672,590 (9.30%)
CPG_t01	41,741,833	40,856,906 (97.88%)	31,256,812 (74.88%)	8,496,891 (20.36%)
CPG_t02	51,075,388	49,629,954 (97.17%)	43,300,839 (84.78%)	4,743,787 (9.29%)
CPG_t03	45,709,720	44,822,951 (98.06%)	39,383,635 (86.16%)	4,289,454 (9.38%)
CPG_t04	58,471,674	57,062,506 (97.59%)	48,200,168 (82.43%)	7,214,612 (12.34%)
CPG_t05	53,201,290	51,887,218 (97.53%)	45,639,584 (85.79%)	4,729,043 (8.89%)
CPG_t06	44,557,837	43,475,081 (97.57%)	38,442,281 (86.28%)	3,803,060 (8.54%)
CPG_t07	54,898,079	53,706,790 (97.83%)	47,214,162 (86.00%)	5,121,203 (9.33%)
CPG_t08	59,475,457	57,899,357 (97.35%)	51,445,657 (86.50%)	4,687,555 (7.88%)
CPG_t09	67,227,645	65,580,567 (97.55%)	57,608,843 (85.69%)	5,975,010 (8.89%)
CPG_t10	41,486,749	40,690,203 (98.08%)	30,974,039 (74.66%)	8,446,625 (20.36%)
CPG_t11	50,429,168	49,112,966 (97.39%)	42,773,437 (84.82%)	4,702,433 (9.32%)
CPG_t12	45,476,232	44,666,755 (98.22%)	39,082,399 (85.94%)	4,279,147 (9.41%)
JNCG_a01	36,056,464	35,241,587 (97.74%)	33,316,019 (92.40%)	1,009,459 (2.80%)
JNCG_a02	37,055,538	36,321,838 (98.02%)	34,168,129 (92.21%)	1,201,262 (3.24%)
JNCG_a03	41,819,588	40,937,194 (97.89%)	38,790,415 (92.76%)	998,144 (2.39%)
JNCG_a04	36,828,005	35,933,084 (97.57%)	33,965,993 (92.23%)	965,701 (2.62%)
JNCG_a05	36,711,692	35,933,404 (97.88%)	33,905,227 (92.36%)	1,055,850 (2.88%)
JNCG_a06	41,937,934	40,964,973 (97.68%)	38,746,536 (92.39%)	1,049,451 (2.50%)
JNCG_a07	38,905,740	37,913,643 (97.45%)	35,903,674 (92.28%)	875,737 (2.25%)
JNCG_a08	38,336,990	37,566,416 (97.99%)	35,566,960 (92.77%)	959,278 (2.50%)
JNCG_c01	43,992,541	42,861,932 (97.43%)	40,592,457 (92.27%)	937,480 (2.13%)
JNCG_c02	36,886,373	36,045,363 (97.72%)	34,128,742 (92.52%)	746,946 (2.02%)
JNCG_c03	36,415,383	35,625,169 (97.83%)	33,729,740 (92.62%)	776,658 (2.13%)
JNCG_t01	41,107,240	40,223,434 (97.85%)	38,045,340 (92.55%)	828,096 (2.01%)
JNCG_t02	45,389,993	44,364,179 (97.74%)	42,135,864 (92.83%)	911,309 (2.01%)
JNCG_t03	41,860,671	40,960,666 (97.85%)	38,916,607 (92.97%)	929,289 (2.22%)

### 3.2 Differential RNA expression analysis

**Figure 2:**
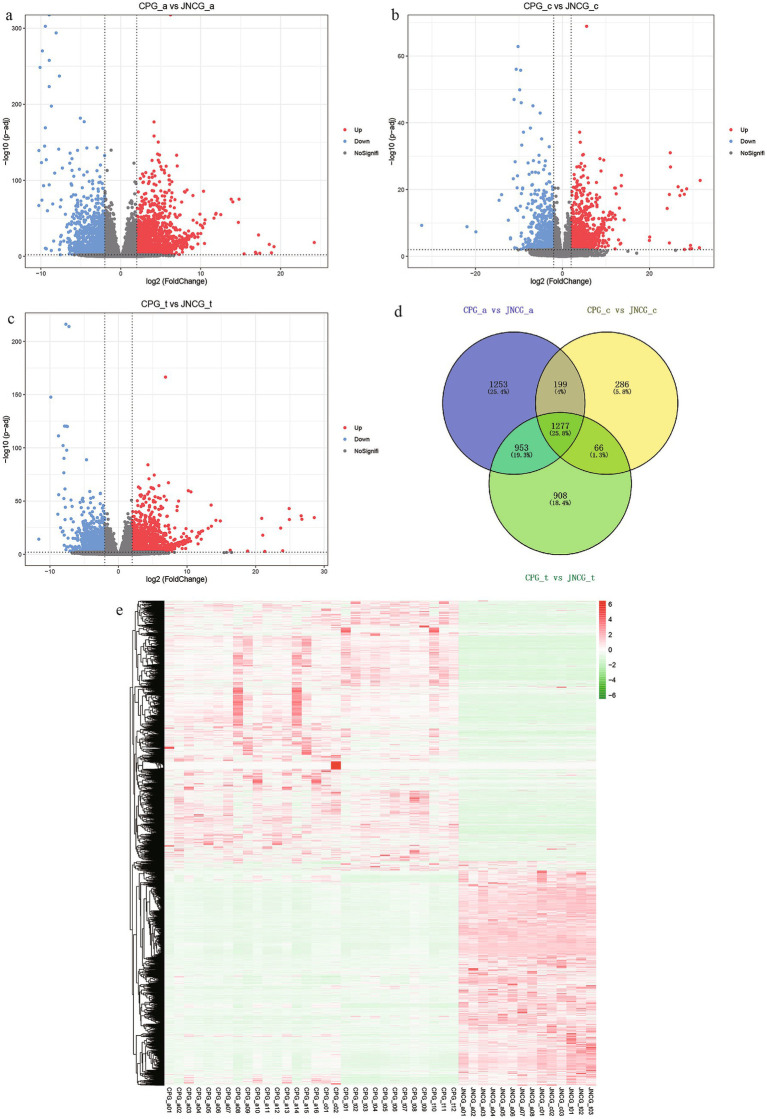
Differential expression results. **(a)** Volcano plot for CPG_a vs. JNCG_a. **(b)** Volcano plot for CPG_c vs. JNCG_c. **(c)** Volcano plot for CPG_t vs. JNCG_t. **(d)** Venn diagram for three phases of differential expression. **(e)** Clustering heatmap of all differentially expressed genes.

Differential expression analysis was performed on the count data of CPG and JNCG for three different growth phases, and volcano plots were generated (Figures 2a–c). The results of differential analysis are presented in Table 3. In the anagen, 2,173 genes were upregulated, and 1,509 genes were downregulated between CPG and JNCG. In the catagen, 1,089 genes were upregulated, and 739 genes were downregulated. In the telogen, 1,808 genes were upregulated, and 1,396 genes were downregulated. Venn analysis (Figure 2d) revealed a total of 4,942 differentially expressed genes, with 1,277 genes differentially expressed in all three phases, 1,218 genes in two phases, and 2,447 genes in one phase only. A clustering heatmap of the FPKM data for all differentially expressed genes (Supplementary Table 2) is shown in Figure 2e, where clear expression differences between the CPG and JNCG groups were observed.

**Table 3:** Differential expression RNA statistical table.

Comparison group	*p*-adj	log2FoldChange	Up	Down	Diff.
CPG_a vs. JNCG_a	0.01	2	2,173	1,509	3,682
CPG_c vs. JNCG_c	0.01	2	1,089	739	1,828
CPG_t vs. JNCG_t	0.01	2	1,808	1,396	3,204

### 3.3 Differential RNA expression analysis

**Figure 3:**
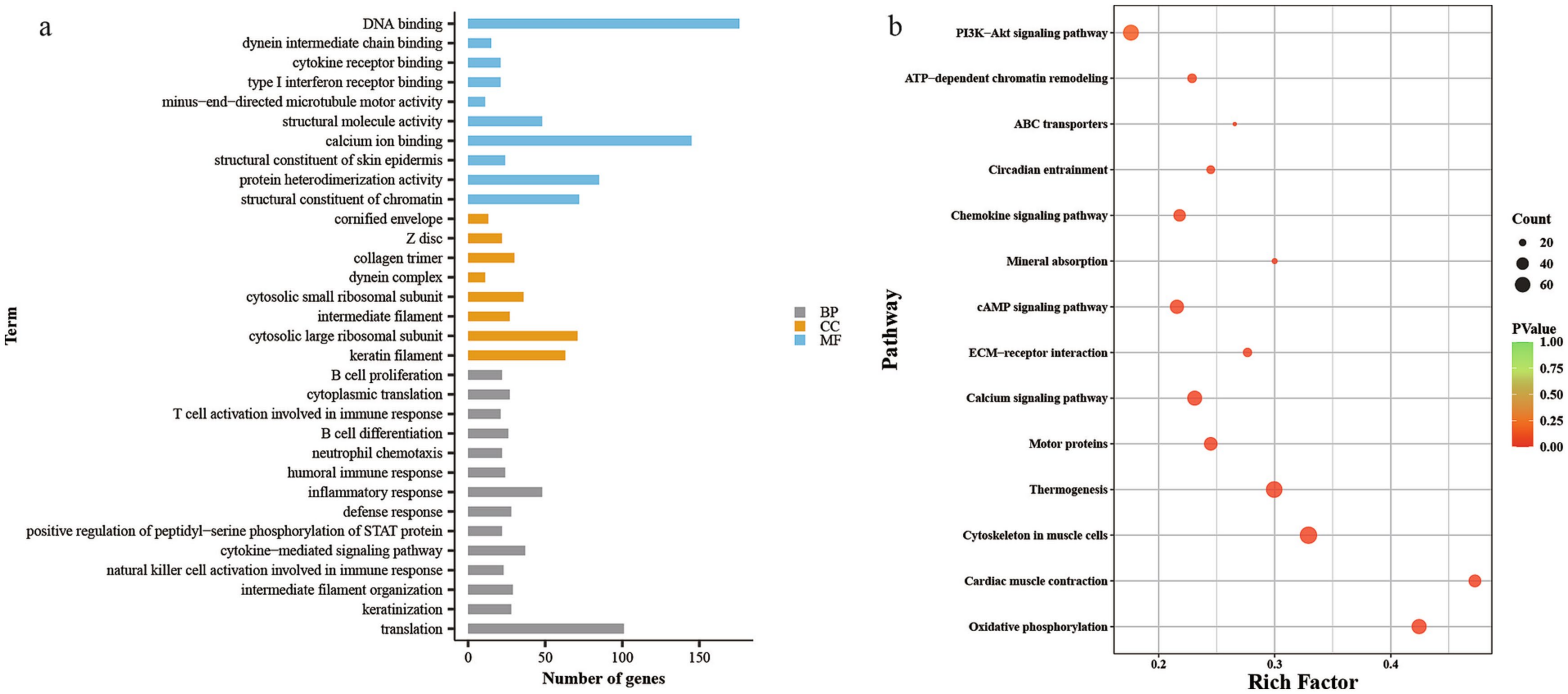
Differential mRNAs GO analysis histogram and KEGG analysis of bubble map. **(a)** GO analysis histogram, **(b)** KEGG analysis of bubble map.

Functional enrichment analysis was performed for the 4,942 differentially expressed genes. GO enrichment analysis (Figure 3a and Supplementary Table 3) showed that 2,418 genes were enriched in molecular function (MF), with 70 MF categories identified, of which 55 were significantly enriched, mainly related to structural constituent of skin epidermis, calcium ion binding, dynein intermediate chain binding, and DNA binding. A total of 2,301 genes were enriched in biological process (BP), with 149 BP categories, 97 of which were significantly enriched, primarily in processes like keratinization, intermediate filament organization, natural killer cell activation in immune response, and inflammatory response. In cell component (CC), 2,584 genes were enriched across 45 CC categories, with 36 significantly enriched, mainly related to keratin filament, intermediate filament, and extracellular space.

KEGG pathway enrichment analysis (Figure 3b and Supplementary Table 4) revealed that 1,396 genes were enriched in 60 KEGG pathways, with 51 pathways significantly enriched. The differentially expressed genes were primarily enriched in pathways related to thermogenesis, PI3K-Akt signaling, ECM-receptor interaction, cAMP signaling, and ABC transporters, all of which have been previously reported in studies of secondary hair follicles.

### 3.4 Keratin and intermediate filament-related GO category analysis

**Figure 4:**
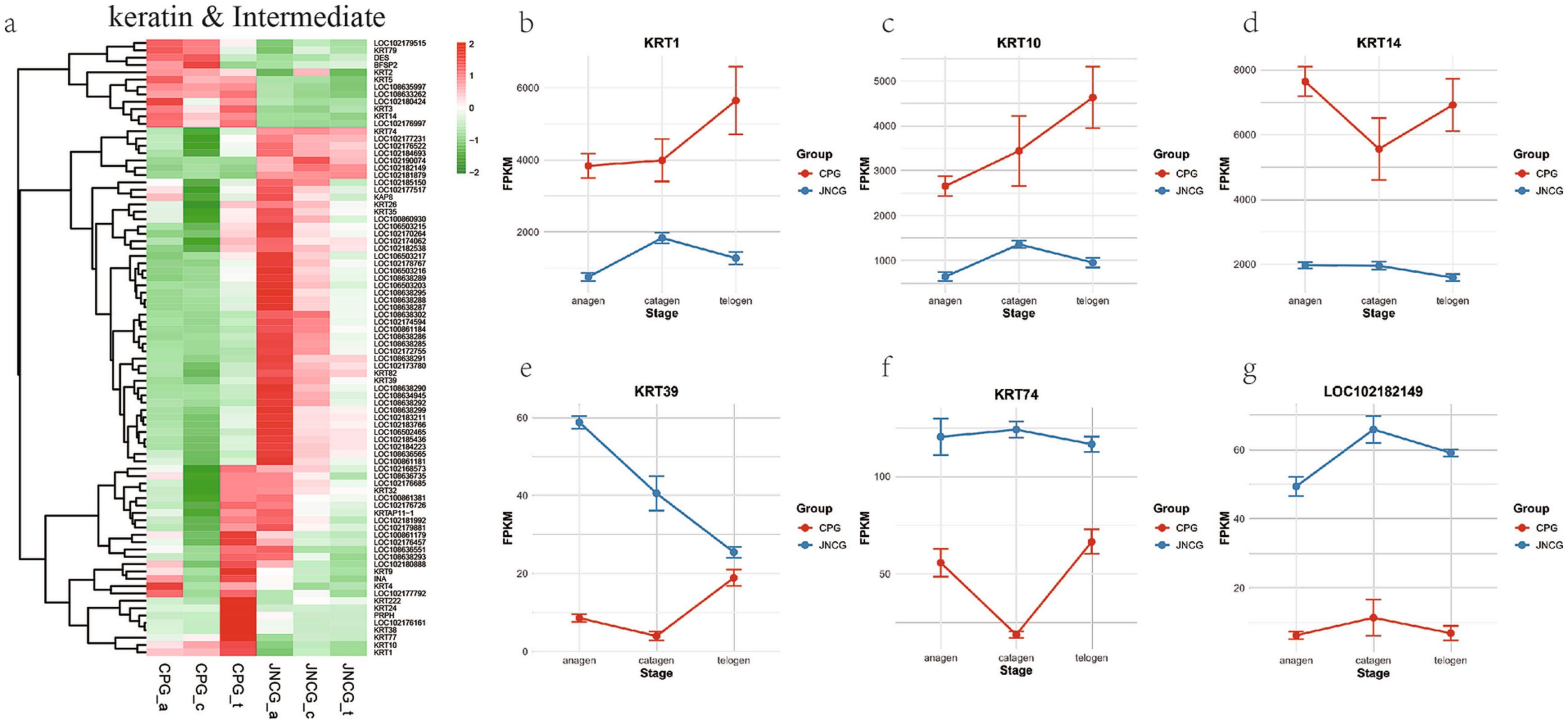
Gene expression analysis for keratin and intermediate filament GO categories. **(a)** Gene clustering heatmap. **(b–g)** Expression trend maps of some key mRNAs.

In GO enrichment analysis, several keratin-related categories were identified in BP and CC, including keratinization (GO:0031424), intermediate filament organization (GO:0045109), keratin filament (GO:0045095), and intermediate filament (GO:0005882), all of which are related to fiber formation in cashmere and hair. Analysis of 84 genes in these categories (Figure 4a) revealed that these genes could be classified into three groups. The first group, with higher expression in CPG than JNCG, included genes like *KRT14*, *BFSP2*, *KRT79*, and *KRT5*. The second group had lower expression in CPG than JNCG, including *KRT74*, *KRT39*, and *LOC102182149* (epiplakin). The third group included genes like *KRT1*, *KRT10*, and *KRT4*. Expression trends of key genes (Figures 4b–g) were analyzed, showing that *KRT1*, *KRT10*, and *KRT14* had higher expression in CPG than in JNCG. *KRT1* and *KRT10* showed lower expression in the anagen and catagen phases in CPG, while in JNCG, their expression was higher in anagen and telogen compared to catagen. *KRT39*, *KRT74*, and *LOC102182149* exhibited higher expression in JNCG compared to CPG, with a trend of decreasing expression in JNCG from anagen to telogen, whereas the trend in CPG was reversed, with rapid upregulation in catagen.

### 3.5 PI3K-Akt signaling pathway analysis

**Figure 5:**
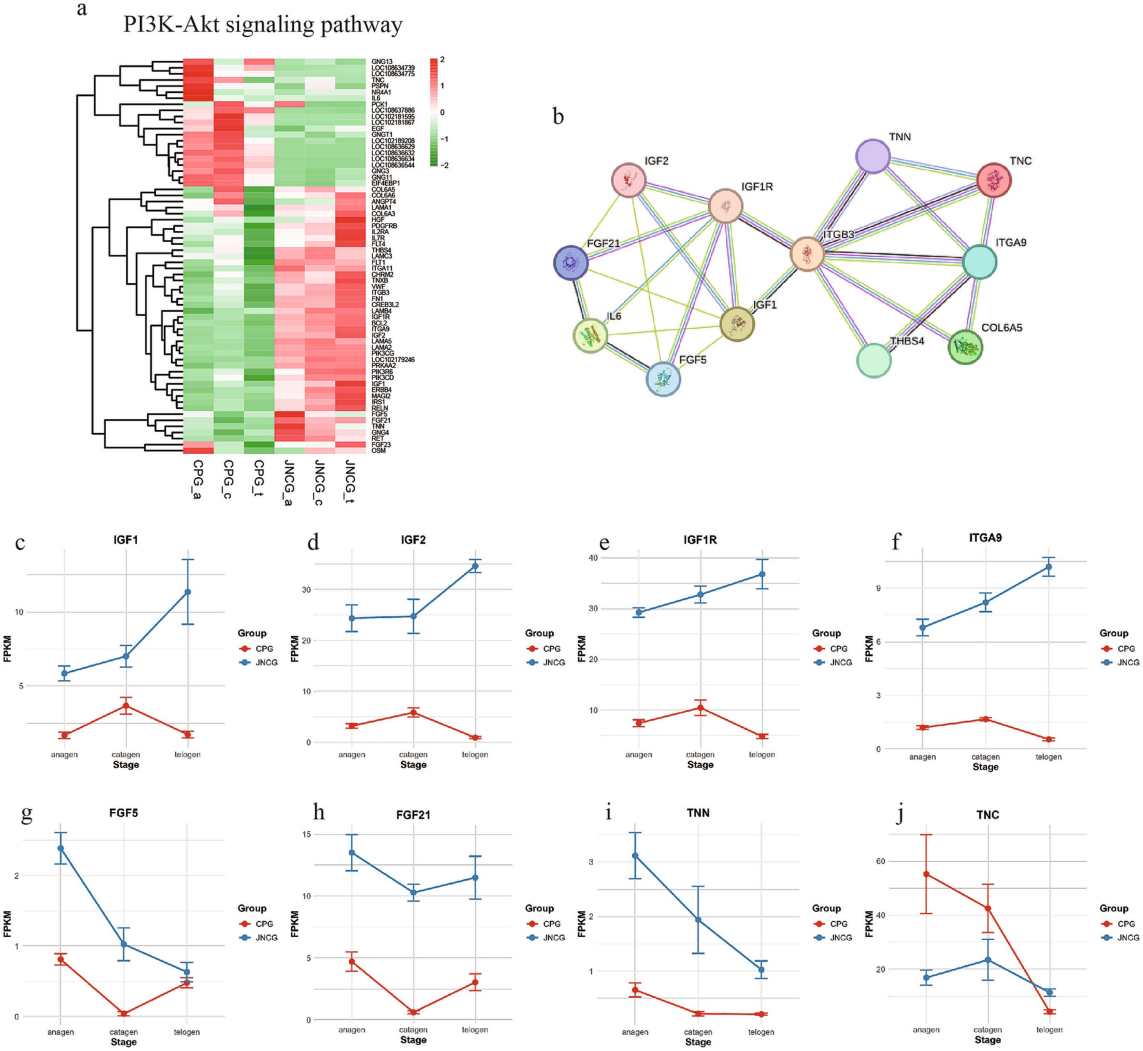
Gene expression analysis in the PI3K-Akt signaling pathway. **(a)** Gene clustering heatmap. **(b)** PPI analysis. **(c-j)** Expression trend maps of some key mRNAs.

The PI3K-Akt signaling pathway is a crucial pathway in secondary hair follicle development, regulating hair growth and quality. Analysis of 65 genes enriched in this pathway (Figure 5a) revealed two distinct gene groups. One group showed higher expression in CPG, including genes like *TNC* and *GNGT1*, while the other group showed higher expression in JNCG, including *IGF1*, *IGF2*, *IFG1R*, *ITGA9*, *FGF5*, *FGF21*, and *TNN*. A PPI network analysis (Figure 5b) showed direct interactions between several proteins, with ITGB3 at the center linking two protein networks. *IGF1*, *IGF2*, *IFG1R*, *FGF5*, and *FGF21* were closely interconnected, while *TNC*, *TNN*, and *ITGA9* formed another tight network. The expression trends for these genes (Figures 5c–j) revealed consistent patterns for *IGF1*, *IGF2*, *IFG1R*, and *ITGA9*, with higher expression levels in JNCG than CPG. In JNCG, expression was higher in telogen compared to anagen and catagen, while in CPG, the highest expression was observed in catagen. The expression trends for *FGF5*, *FGF21*, and TNN were similar, with higher levels in JNCG than in CPG, with the highest expression of *FGF5* in anagen in both groups.

### 3.6 Thermogenesis pathway analysis

**Figure 6:**
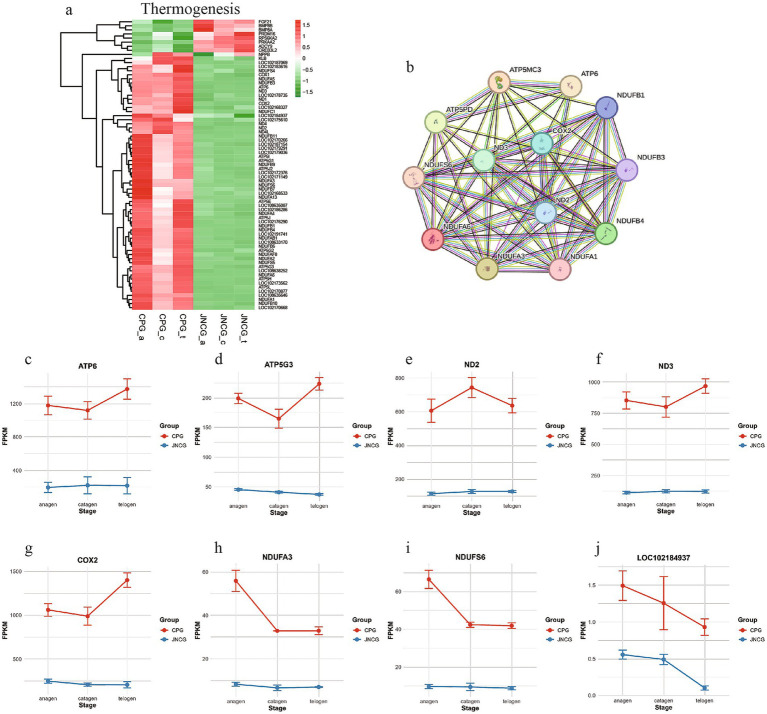
Gene expression analysis in thermogenesis. **(a)** Gene clustering heatmap. **(b)** PPI analysis. **(c–j)** Expression trend maps of some key mRNAs.

Thermogenesis is associated with heat production, and the production of cashmere in goats is also aimed at protecting against cold. Different breeds of goats exhibit distinct cashmere production mechanisms due to environmental and coat differences. Analysis of 71 genes enriched in this pathway (Figure 6a) revealed two main groups. One group showed higher expression in CPG, including genes such as *ATP6*, *ND2*, *ND3*, and *COX2*, which are related to energy metabolism, indicating that CPG, living in a colder environment, may have upregulated thermogenesis-related genes. The other group showed higher expression in JNCG, including *FGF21* and *BMP8B*. PPI network analysis (Figure 6b) revealed strong interactions between proteins in this pathway. Expression trends of selected genes (Figures 6c–j) indicated higher expression of *ATP6*, *ATP5G3*, *ND3*, and *COX2* in CPG, with the highest expression observed in telogen. JNCG showed lower expression for these genes. *NDUFA3* and *NDUFS6* exhibited the highest expression in CPG during anagen. *LOC102184937* (cytochrome c oxidase subunit 6B2) showed higher expression in CPG, with both CPG and JNCG showing the highest expression in anagen.

### 3.7 Pathway network and key gene analysis

**Figure 7:**
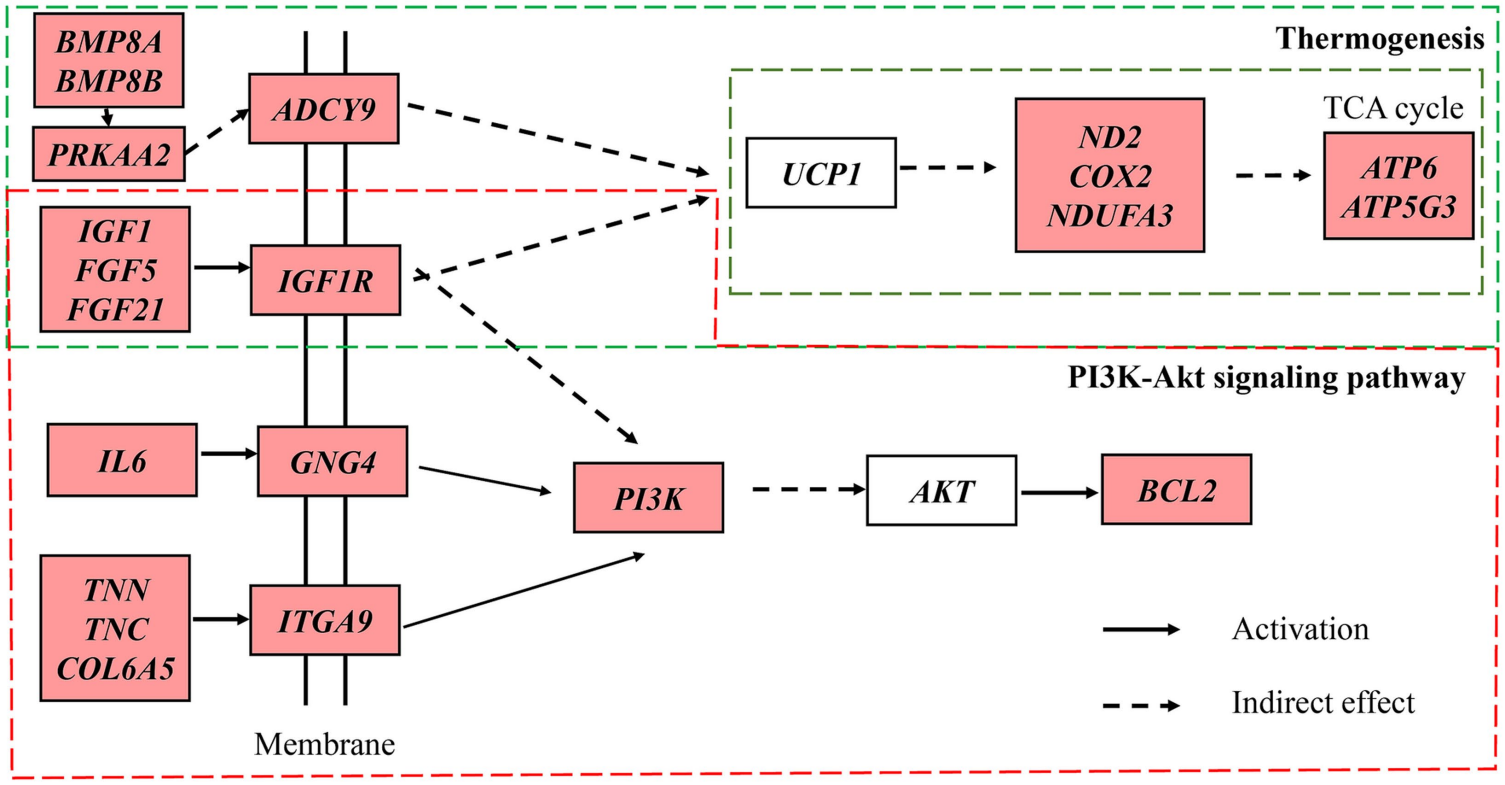
Interactive network control chart.

Based on the analysis of key pathways and entries, and combining research on secondary hair follicle development, a selection of key candidate genes was identified (Table 4). These genes exhibited differential expression in the skin tissues of both cashmere goat breeds and at different stages of secondary hair follicle growth. Two key pathways, the PI3K-Akt signaling pathway and thermogenesis, were selected for further analysis, as these pathways are interrelated. Using the KEGG network, we constructed a KEGG molecular network diagram for the key genes (Figure 7). The diagram shows that several important molecules, upstream of these pathways (such as *BMP8B*, *IFG1R*, *FGF5*, *FGF21*, *TNN*, etc.), regulate corresponding receptor genes (such as *ADCY9*, *IGF1R*, *GNG4*, and *ITGA9*). Through indirect or direct interactions, these genes influence the *PI3K* and *UCP1* genes, which in turn regulate *ND2*, *COX2*, *ATP6*, and other genes to maintain the TCA cycle. The *PI3K* gene further regulates the *AKT* and *BCL2* genes.

**Table 4:** Candidate gene information table.

Gene_name	CPG_a	CPG_c	CPG_t	JNCG_a	JNCG_c	JNCG_t	KEGG & GO term
*IGF1*	1.67 ± 0.89	3.67 ± 0.8	1.73 ± 0.74	5.85 ± 1.42	7.01 ± 1.27	11.35 ± 3.79	PI3K-Akt signaling pathway
*TNN*	0.66 ± 0.51	0.22 ± 0.05	0.21 ± 0.07	3.11 ± 1.2	1.94 ± 1.07	1.03 ± 0.28	PI3K-Akt signaling pathway
*IGF1R*	7.44 ± 2.61	10.47 ± 2.15	4.82 ± 1.47	29.2 ± 2.66	32.73 ± 2.91	36.74 ± 5.03	PI3K-Akt signaling pathway
*TNC*	55.21 ± 58.37	42.49 ± 12.68	4.23 ± 2.52	16.87 ± 7.83	23.48 ± 13.02	11.37 ± 2.36	PI4K-Akt signaling pathway
*ITGA9*	1.2 ± 0.42	1.68 ± 0.13	0.55 ± 0.28	6.79 ± 1.32	8.2 ± 0.91	10.19 ± 0.91	PI5K-Akt signaling pathway
*FGF21*	4.72 ± 3.05	0.63 ± 0.18	3.05 ± 2.34	13.51 ± 4.14	10.29 ± 1.17	11.5 ± 2.99	PI3K-Akt signaling pathway; thermogenesis
*IGF2*	3.22 ± 1.76	5.86 ± 1.27	0.93 ± 0.71	24.35 ± 7.39	24.74 ± 5.78	34.61 ± 2.27	PI3K-Akt signaling pathway
*FGF5*	0.81 ± 0.33	0.04 ± 0.04	0.48 ± 0.24	2.39 ± 0.63	1.03 ± 0.4	0.63 ± 0.24	PI3K-Akt signaling pathway
*ND2*	606.28 ± 277.04	743.55 ± 84.39	636.14 ± 148.72	113.68 ± 27.04	126.61 ± 20.13	126.62 ± 9.67	Thermogenesis
*COX2*	1060.91 ± 283.88	989.21 ± 145.68	1399.65 ± 284.13	248.88 ± 67.96	209.5 ± 29.33	208.2 ± 62.67	Thermogenesis
*LOC102184937*	1.5 ± 0.81	1.26 ± 0.51	0.93 ± 0.39	0.56 ± 0.17	0.49 ± 0.12	0.1 ± 0.05	Thermogenesis
*ATP5G3*	199.04 ± 35.13	164.93 ± 22.51	223.58 ± 36.89	45.4 ± 3.81	40.97 ± 2.72	37.19 ± 2.41	Thermogenesis
*NDUFA3*	55.94 ± 19.61	32.87 ± 0.15	32.91 ± 6.25	8.28 ± 2.49	6.6 ± 2.26	6.97 ± 0.28	Thermogenesis
*NDUFS6*	66.47 ± 19.46	42.4 ± 2	41.88 ± 5.28	9.61 ± 3.07	9.4 ± 3.42	8.73 ± 1.41	Thermogenesis
*ATP6*	1179.27 ± 442.67	1119.93 ± 149.87	1376.14 ± 422.64	195.33 ± 174.81	220.05 ± 176.31	216.16 ± 171.58	Thermogenesis
*ND3*	853.98 ± 276.31	802.04 ± 116.26	969.15 ± 201.05	112.72 ± 28.84	123.64 ± 22.79	122.16 ± 22.27	Thermogenesis
*ATP5H*	225.34 ± 65.45	160.08 ± 13.89	225.04 ± 33.69	45.12 ± 6.01	46.54 ± 4.94	43.86 ± 3.16	Thermogenesis
*KRT10*	2658.95 ± 889.51	3443.59 ± 1110.07	4634.39 ± 2374.12	641.18 ± 281.83	1362.35 ± 138.2	954.03 ± 187.6	Keratin filament
*KRT14*	7639.19 ± 1833.48	5555.74 ± 1354.56	6917.44 ± 2788.93	1964.26 ± 272.83	1951.58 ± 221.14	1583.47 ± 185.9	Keratin filament; intermediate filament
*KRT1*	3838.54 ± 1361.05	3989.69 ± 828.35	5643.65 ± 3253.3	757.59 ± 324.03	1838.78 ± 259.62	1279.9 ± 292.36	Keratin filament
*LOC102182149*	6.33 ± 4.28	11.4 ± 7.38	6.93 ± 7.31	49.38 ± 7.92	65.84 ± 6.76	59.08 ± 1.78	Keratin filament
*KRT74*	55.72 ± 28.68	18.8 ± 2.38	66.5 ± 22.07	120.35 ± 26.57	124.03 ± 7.21	116.54 ± 6.77	Keratin filament
*KRT39*	8.58 ± 4	3.96 ± 1.62	18.89 ± 7.3	58.77 ± 4.62	40.54 ± 7.66	25.46 ± 2.46	Intermediate filament
*LOC102181879*	13.49 ± 6.8	17.13 ± 3.73	6.01 ± 4.73	105.49 ± 10.03	108.14 ± 20.68	110.55 ± 6.5	Intermediate filament

## 4 Discussion

This study, through a comparative analysis of the skin transcriptomes of JNCG and CPG, reveals differences in gene expression across different follicle growth stages. These differences provide a molecular basis for understanding the variations in cashmere quality among different breeds of cashmere goats.

The secondary hair follicles of cashmere goats produce cashmere primarily to withstand cold environments. The cashmere layer in the skin efficiently helps cashmere goats maintain body temperature. Numerous studies have shown that cashmere growth is influenced by multiple factors, including breed, genetics, nutrition, age, shearing, and environment (26–29). Several studies have demonstrated that signaling pathways such as Wnt, TGF-β, PI3K-Akt, and cAMP regulate the development of secondary follicles and cashmere growth in cashmere goats (6, 30). In this study, differentially expressed genes were also enriched in the PI3K-Akt and cAMP signaling pathways, indicating that the variations in cashmere between these two breeds are influenced by these pathways, which play important roles in follicle development and cashmere fiber growth.

The PI3K-Akt signaling pathway is critical in regulating cell growth, differentiation, and survival. Among its key regulators, *IGF1* is upregulated, promoting cell proliferation and facilitating the transition of SHFs dermal cells in Inner Mongolia cashmere goats from the anagen to telogen (31). Additionally, *FGF5* overexpression can upregulate *IGF1*, and *FGF5* itself acts as an inhibitor of hair growth (32, 33). Downregulation of *FGF5* can influence the PI3K-Akt signaling pathway, and studies by Hu et al. (34) demonstrated that knocking in the *VEGF* gene at the *FGF5* locus through gene editing led to increased cashmere yield and fiber length in cashmere goats. In our study, *FGF5* expression in CPG was lower than in JNCG, further confirming that *FGF5* regulates cashmere quality. Similarly, *FGF21* influences the hair length of cashmere goats, showing a significant positive correlation with hair length (35). *FGF21* knockout mice exhibit reduced body weight, slower hair growth, smaller follicle diameters, decreased follicle numbers, and lower hair density (36, 37). The differential expression patterns of *IGF1*, *IGF1R*, *FGF5*, and *FGF21* in the skin tissues of the two cashmere goat breeds suggest that these growth factors play important roles in cashmere fiber growth and quality formation.

Additionally, we found that differentially expressed genes were significantly enriched in the thermogenesis pathway. Research by Nocelli et al. (9) showed that thermogenesis influences the transition from the growth phase to the regression phase in secondary follicles of Italian cashmere goats. Thermogenesis reflects the differences in environmental adaptation between these two cashmere goat breeds. JNCG is primarily distributed in the Aksu region of Xinjiang, China, where the environment is characterized by arid, semi-arid conditions and shrub grasslands. In contrast, CPG inhabit the Ladakh region of India, a high-altitude area exceeding 3,000 meters above sea level. This region is cold, has thin air with lower oxygen levels, and experiences extremely low temperatures in winter. The higher expression levels of thermogenesis-related genes in CPG may be associated with its adaptation to cold, high-altitude environments, where more efficient energy metabolism is required to maintain body temperature and physiological functions. In studies involving humans and mice, *COX2* inhibitors have been shown to restore hair growth, indicating that hair loss is attributed to elevated *COX2* enzyme activity (38). Transgenic mice overexpressing *COX2* exhibit hair loss, and feeding them *COX2* inhibitors suppresses *COX2* activity and halts hair loss progression (39). Zhang et al. (40) investigated hedgehog spine follicles and mouse hair follicle development, revealing that *COX2* acts upstream of ATP synthase, influencing energy metabolism and cell proliferation to regulate spine follicle size. In this study, *COX2* and numerous ATP-related differentially expressed genes were also identified in both cashmere goat breeds. These genes exhibited higher expression levels in CPG, which may correlate with the unstable cashmere yield observed in this breed.

Furthermore, a large number of KRTs, such as *KRT10*, *KRT14*, *KRT39*, and *KRT74*, were identified among the differentially expressed genes. KRT10 protein content in cashmere fibers influences fiber diameter, with higher expression levels observed in coarser cashmere compared to finer cashmere (41), and *KRT10* gene expression is significantly higher in fine wool Angora rabbits (42). In our study, KRT10 expression in CPG was significantly higher than in JNCG, potentially due to the undefined cashmere fineness of the sampled JNCG individuals, which exhibited finer fibers. Additionally, *KRT39* and *KRT74* are highly expressed in long-haired cashmere goats and play a role in regulating hair growth (43). *KRT74* expression in adult fine-wool sheep is more than twice as high as in adult coarse-wool sheep (44). In our study, *KRT74* expression in JNCG was over twice that of CPG, further supporting its influence on fiber fineness and aligning with previous findings that JNCG produces finer cashmere fibers. These results highlight the critical role of keratin genes in fiber formation and hair structure development.

## 5 Conclusion

This study systematically analyzed the transcriptome data of JNCG and CPG skin, revealing gene expression differences in the skin tissues of the two cashmere goat breeds. The findings indicate that the PI3K-Akt signaling pathway, thermogenesis pathway, and keratin-related genes play important roles in the development of secondary hair follicles and the formation of cashmere fiber quality. A total of 24 key candidate genes, including *IGF1*, *IGF1R*, *FGF5*, *FGF21*, *ND2*, *COX2*, *KRT10*, *KRT39*, and *KRT74*, were identified, providing a theoretical basis for the future genetic improvement of cashmere goats.

## Data Availability

RNA-Seq data are from PRJNA688899, PRJNA480975, and PRJNA778726. The FPKM data of differentially expressed genes are shown in Supplementary Table 2. Please contact the corresponding author for other data (Gao Gong: ggao1995@163.com).

## Footnotes

^1^ https://cn.string-db.org/cgi/input.pl

## Supplementary material

SUPPLEMENTARY TABLE 1 SRA data download statistics table.

SUPPLEMENTARY TABLE 2 FPKM data for all differentially expressed genes.

SUPPLEMENTARY TABLE 3 GO analysis table of differentially expressed genes.

SUPPLEMENTARY TABLE 4 KEGG enrichment analysis table of differentially expressed genes.

SUPPLEMENTARY MATERIAL 1 Linux scripts and Python code.
