# Supplementary material for: Molecular mechanisms underlying cashmere quality differences between Jiangnan cashmere goats and Changthangi pashmina goats
Source: Front Vet Sci. 2025 May 28;12:1571803. doi: 10.3389/fvets.2025.1571803 (PMC12153449; doi:10.3389/fvets.2025.1571803)
Supplement: SUPPLEMENTARY MATERIAL 1 — Linux scripts and Python code. [file Table_5.docx]

#Build index

#hisat2

hisat2_extract_splice_sites.py /home/gonggao/reference/data/GCF_001704415.1_ARS1_genomic.gtf > genome.ss

hisat2_extract_exons.py /home/gonggao/reference/data/GCF_001704415.1_ARS1_genomic.gtf > genome.exon

nohup hisat2-build -p 50 --ss /home/gonggao/reference/ARS1_hisat2/genome.ss --exon /home/gonggao/reference/ARS1_hisat2/genome.exon /home/gonggao/reference/data/GCF_001704415.1_ARS1_genomic.fna ARS1 > hisat2-build.log &

#Data quality control

while read id; do

trimmomatic PE -phred33 \

"${id}_1.fastq.gz" "${id}_2.fastq.gz" \

"${id}_1.clean.fastq.gz" /dev/null \

"${id}_2.clean.fastq.gz" /dev/null \

ILLUMINACLIP:TruSeq3-PE.fa:2:30:10 \

LEADING:3 TRAILING:3 \

SLIDINGWINDOW:4:15 MINLEN:36 \

> "${id}.log" 2>&1 &

done < fastq1-id.txt

#Alignment, strand-specific

while read id; do

hisat2 -p 4 --dta --phred33 --no-unal -t --rna-strandness RF \

--un-conc-gz ${id}.unmapped.fastq.gz \

--summary-file ${id}_mapping_rate.xls \

-x /home/gonggao/reference/ARS1_hisat2/ARS1 \

-1 ${id}_1.clean.fastq.gz \

-2 ${id}_2.clean.fastq.gz | \

samtools sort -O bam -@ 8 -o ${id}.bam > "${id}.log" 2>&1 &

done < fastq1a.txt

#Alignment, mRNA

while read id; do

hisat2 -p 4 --dta --phred33 --no-unal -t \

--un-conc-gz ${id}.unmapped.fastq.gz \

--summary-file ${id}_mapping_rate.xls \

-x /home/gonggao/reference/ARS1_hisat2/ARS1 \

-1 ${id}_1.clean.fastq.gz \

-2 ${id}_2.clean.fastq.gz | \

samtools sort -O bam -@ 8 -o ${id}.bam > "${id}.log" 2>&1 &

done < fastq1a.txt

#Quantification, strand-specific

cat list.bam |cut -d"." -f 1|sort -u|while read id; do

stringtie -e -B --rf -p 50 -G /data01/public/reference/ARS1/GCF_001704415.1_ARS1_genomic.gff -o ./${id}/${id}.stringtie_o.gtf -A ./${id}/${id}_o.tab ../../${id}.hisat2.bam;

done

#Quantification, mRNA

cat list.bam |cut -d"." -f 1|sort -u|while read id; do

stringtie -e -B -p 50 -G /data01/public/reference/ARS1/GCF_001704415.1_ARS1_genomic.gff -o ./${id}/${id}.stringtie_o.gtf -A ./${id}/${id}_o.tab ../../${id}.hisat2.bam;

done

#Run quantification script

python getCOUNT.py -i /home/gonggao/goat_rna/03.hisat2/code_rna/a/

python getFPKM.py -i /home/gonggao/goat_rna/03.hisat2/code_rna/a/

#getCOUNT.py

#!/usr/bin/env python2

import re, csv, sys, os, glob, warnings, itertools

from math import ceil

from optparse import OptionParser

from operator import itemgetter

MIN_PYTHON = (2, 7)

if sys.version_info < MIN_PYTHON:

sys.exit("Python %s.%s or later is required.\n" % MIN_PYTHON)

parser=OptionParser(description='Generates two CSV files containing the count matrices for genes and transcripts, using the coverage values found in the output of `stringtie -e`')

parser.add_option('-i', '--input', '--in', default='.', help="a folder containing all sample sub-directories, or a text file with sample ID and path to its GTF file on each line [default: %default/]")

parser.add_option('-g', default='gene_count_matrix.csv', help="where to output the gene count matrix [default: %default")

parser.add_option('-t', default='transcript_count_matrix.csv', help="where to output the transcript count matrix [default: %default]")

parser.add_option('-l', '--length', default=75, type='int', help="the average read length [default: %default]")

parser.add_option('-p', '--pattern', default=".", help="a regular expression that selects the sample subdirectories")

parser.add_option('-c', '--cluster', action="store_true", help="whether to cluster genes that overlap with different gene IDs, ignoring ones with geneID pattern (see below)")

parser.add_option('-s', '--string', default="MSTRG", help="if a different prefix is used for geneIDs assigned by StringTie [default: %default]")

parser.add_option('-k', '--key', default="prepG", help="if clustering, what prefix to use for geneIDs assigned by this script [default: %default]")

parser.add_option('-v', action="store_true", help="enable verbose processing")

parser.add_option('--legend', default="legend.csv", help="if clustering, where to output the legend file mapping transcripts to assigned geneIDs [default: %default]")

(opts, args)=parser.parse_args()

samples = [] # List of tuples. If sample list, (first column, path). Else, (subdirectory name, path to gtf file in subdirectory)

if (os.path.isfile(opts.input)):

# gtfList = True

try:

fin = open(opts.input, 'r')

for line in fin:

if line[0] != '#':

lineLst = tuple(line.strip().split(None,2))

if (len(lineLst) != 2):

print "Error: line should have a sample ID and a file path:\n%s" % (line.strip())

exit(1)

if lineLst[0] in samples:

print "Error: non-unique sample ID (%s)" % (lineLst[0])

exit(1)

if not os.path.isfile(lineLst[1]):

print "Error: GTF file not found (%s)" % (lineLst[1])

exit(1)

samples.append(lineLst)

except IOError:

print "Error: List of .gtf files, %s, doesn't exist" % (opts.input)

exit(1)

else:

# gtfList = False

## Check that opts.input directory exists

if not os.path.isdir(opts.input):

parser.print_help()

print " "

print "Error: sub-directory '%s' not found!" % (opts.input)

sys.exit(1)

#####

## Collect all samples file paths and if empty print help message and quit

#####

samples = [(i,glob.iglob(os.path.join(opts.input,i,"*.gtf")).next()) for i in next(os.walk(opts.input))[1] if re.search(opts.pattern,i)]

if len(samples) == 0:

parser.print_help()

print " "

print "Error: no GTF files found under base directory %s !" % (opts.input)

sys.exit(1)

RE_GENE_ID=re.compile('gene_id "([^"]+)"')

RE_GENE_NAME=re.compile('gene_name "([^"]+)"')

RE_TRANSCRIPT_ID=re.compile('transcript_id "([^"]+)"')

RE_COVERAGE=re.compile('cov "([\-\+\d\.]+)"')

RE_STRING=re.compile(re.escape(opts.string))

RE_GFILE=re.compile('\-G\s*(\S+)') #assume filepath without spaces..

#####

## Sort the sample names by the sample ID

#####

samples.sort()

#if opts.v:

# print "Sample GTFs found:"

# for s in samples:

# print s[1]

#####

## Checks whether a given row is a transcript

## other options: ex. exon, transcript, mRNA, 5'UTR

#####

def is_transcript(x):

return len(x)>2 and x[2]=="transcript"

def getGeneID(s, ctg, tid):

r=RE_GENE_ID.search(s)

#if r: return r.group(1)

rn=RE_GENE_NAME.search(s)

#if rn: return ctg+'|'+rn.group(1)

if r:

if rn:

return r.group(1)+'|'+rn.group(1)

else:

return r.group(1)

return tid

def getCov(s):

r=RE_COVERAGE.search(s)

if r:

v=float(r.group(1))

if v<0.0: v=0.0

return v

return 0.0

def is_overlap(x,y): #NEEDS TO BE INTS!

return x[0]<=y[1] and y[0]<=x[1]

def t_overlap(t1, t2): #from badGenes: chromosome, strand, cluster, start, end, (e1start, e1end)...

if t1[0] != t2[0] or t1[1] != t2[1] or t1[5]<t2[4]: return False

for i in range(6, len(t1)):

for j in range(6, len(t2)):

if is_overlap(t1[i], t2[j]): return True

return False

## Average Readlength

read_len=opts.length

## Variables/Matrices to store t/g_counts

t_count_matrix, g_count_matrix=[],[]

##Get ready for clustering, stuff is once for all samples##

geneIDs={} #key=transcript, value=cluster/gene_id

## For each of the sorted sample paths

for s in samples:

badGenes=[] #list of bad genes (just ones that aren't MSTRG)

try:

## opts.input = parent directory of sample subdirectories

## s = sample currently iterating through

## os.path.join(opts.input,s,"*.gtf") path to current sample's GTF

## split = list of lists: [[chromosome, ...],...]

#with open(glob.iglob(os.path.join(opts.input,s,"*.gtf")).next()) as f:

# split=[l.split('\t') for l in f.readlines()]

# if not gtfList:

# f = open(glob.iglob(os.path.join(opts.input,s[1],"*.gtf")).next())

# else:

# f = open(s[1])

with open(s[1]) as f:

split=[l.split('\t') for l in f.readlines()]

## i = numLine; v = corresponding i-th GTF row

for i,v in enumerate(split):

if is_transcript(v):

t_id=RE_TRANSCRIPT_ID.search(v[8]).group(1)

try:

g_id=getGeneID(v[8], v[0], t_id)

except:

print "Problem parsing file %s at line:\n:%s\n" % (s[1], v)

sys.exit(1)

geneIDs.setdefault(t_id, g_id)

if not RE_STRING.match(g_id):

badGenes.append([v[0],v[6], t_id, g_id, min(int(v[3]),int(v[4])), max(int(v[3]),int(v[4]))]) #chromosome, strand, cluster/transcript id, start, end

j=i+1

while j<len(split) and split[j][2]=="exon":

badGenes[len(badGenes)-1].append((min(int(split[j][3]), int(split[j][4])), max(int(split[j][3]), int(split[j][4]))))

j+=1

except StopIteration:

warnings.warn("Didn't get a GTF in that directory. Looking in another...")

else: #we found the "bad" genes!

break

##THE CLUSTERING BEGINS!##

if opts.cluster and len(badGenes)>0:

clusters=[] #lists of lists (could be sets) or something of transcripts

badGenes.sort(key=itemgetter(3)) #sort by start coord...?

i=0

while i<len(badGenes): #rather un-pythonic

temp_cluster=[badGenes[i]]

k=0

while k<len(temp_cluster):

j=i+1

while j<len(badGenes):

if t_overlap(temp_cluster[k], badGenes[j]):

temp_cluster.append(badGenes[j])

del badGenes[j]

else:

j+=1

k+=1

if len(temp_cluster)>1:

clusters.append([t[2] for t in temp_cluster])

i+=1

print len(clusters)

for c in clusters:

c.sort()

clusters.sort(key=itemgetter(0))

legend=[]

for u,c in enumerate(clusters):

my_ID=opts.key+str((u+1))

legend.append(list(itertools.chain.from_iterable([[my_ID],c]))) #my_ID, clustered transcript IDs

for t in c:

geneIDs[t]=my_ID

## geneIDs[t]="|".join(c) #duct-tape transcript IDs together, disregarding ref_gene_names and things like that

with open(opts.legend, 'w') as l_file:

my_writer=csv.writer(l_file)

my_writer.writerows(legend)

geneDict={} #key=gene/cluster, value=dictionary with key=sample, value=summed counts

t_dict={}

guidesFile='' # file given with -G for the 1st sample

for q, s in enumerate(samples):

if opts.v:

print ">processing sample %s from file %s" % s

lno=0

try:

#with open(glob.iglob(os.path.join(opts.input,s,"*.gtf")).next()) as f: #grabs first .gtf file it finds inside the sample subdirectory

# if not gtfList:

# f = open(glob.iglob(os.path.join(opts.input,s[1],"*.gtf")).next())

# else:

f = open(s[1])

transcript_len=0

for l in f:

lno+=1

if l.startswith('#'):

if lno==1:

ei=l.find('-e')

if ei<0:

print "Error: sample file %s was not generated with -e option!" % ( s[1] )

sys.exit(1)

gf=RE_GFILE.search(l)

if gf:

gfile=gf.group(1)

if guidesFile:

if gfile != guidesFile:

print "Warning: sample file %s generated with a different -G file (%s) than the first sample (%s)" % ( s[1], gfile, guidesFile )

else:

guidesFile=gfile

else:

print "Error: sample %s was not processed with -G option!" % ( s[1] )

sys.exit(1)

continue

v=l.split('\t')

if v[2]=="transcript":

if transcript_len>0:

## transcriptList.append((g_id, t_id, int(ceil(coverage*transcript_len/read_len))))

t_dict.setdefault(t_id, {})

t_dict[t_id].setdefault(s[0], int(ceil(coverage*transcript_len/read_len)))

t_id=RE_TRANSCRIPT_ID.search(v[len(v)-1]).group(1)

#g_id=RE_GENE_ID.search(v[len(v)-1]).group(1)

g_id=getGeneID(v[8], v[0], t_id)

#coverage=float(RE_COVERAGE.search(v[len(v)-1]).group(1))

coverage=getCov(v[8])

transcript_len=0

if v[2]=="exon":

transcript_len+=int(v[4])-int(v[3])+1 #because end coordinates are inclusive in GTF

## transcriptList.append((g_id, t_id, int(ceil(coverage*transcript_len/read_len))))

t_dict.setdefault(t_id, {})

t_dict[t_id].setdefault(s[0], int(ceil(coverage*transcript_len/read_len)))

except StopIteration:

# if not gtfList:

# warnings.warn("No GTF file found in " + os.path.join(opts.input,s[1]))

# else:

warnings.warn("No GTF file found in " + s[1])

## transcriptList.sort(key=lambda bla: bla[1]) #gene_id

for i,v in t_dict.iteritems():

## print i,v

try:

geneDict.setdefault(geneIDs[i],{}) #gene_id

geneDict[geneIDs[i]].setdefault(s[0],0)

geneDict[geneIDs[i]][s[0]]+=v[s[0]]

except KeyError:

print "Error: could not locate transcript %s entry for sample %s" % ( i, s[0] )

raise

if opts.v:

print "..writing %s " % ( opts.t )

with open(opts.t, 'w') as csvfile:

my_writer = csv.DictWriter(csvfile, fieldnames = ["transcript_id"] + [x for x,y in samples])

my_writer.writerow(dict((fn,fn) for fn in my_writer.fieldnames))

for i in t_dict:

t_dict[i]["transcript_id"] = i

my_writer.writerow(t_dict[i])

if opts.v:

print "..writing %s " % ( opts.g )

with open(opts.g, 'w') as csvfile:

my_writer = csv.DictWriter(csvfile, fieldnames = ["gene_id"] + [x for x,y in samples])

## my_writer.writerow([""]+samples)

## my_writer.writerows(geneDict)

my_writer.writerow(dict((fn,fn) for fn in my_writer.fieldnames))

for i in geneDict:

geneDict[i]["gene_id"] = i #add gene_id to row

my_writer.writerow(geneDict[i])

if opts.v:

print "All done."

#getFPKM.py

#!/usr/bin/env python2

import re, csv, sys, os, glob, warnings, itertools

from math import ceil

from optparse import OptionParser

from operator import itemgetter

MIN_PYTHON = (2, 7)

if sys.version_info < MIN_PYTHON:

sys.exit("Python %s.%s or later is required.\n" % MIN_PYTHON)

parser=OptionParser(description='Generates two CSV files containing the count matrices for genes and transcripts, using the coverage values found in the output of `stringtie -e`')

parser.add_option('-i', '--input', '--in', default='.', help="a folder containing all sample sub-directories, or a text file with sample ID and path to its GTF file on each line [default: %default/]")

parser.add_option('-g', default='gene_fpkm_matrix.csv', help="where to output the gene fpkm matrix [default: %default")

parser.add_option('-t', default='transcript_fpkm_matrix.csv', help="where to output the transcript fpkm matrix [default: %default]")

parser.add_option('-l', '--length', default=75, type='int', help="the average read length [default: %default]")

parser.add_option('-p', '--pattern', default=".", help="a regular expression that selects the sample subdirectories")

parser.add_option('-c', '--cluster', action="store_true", help="whether to cluster genes that overlap with different gene IDs, ignoring ones with geneID pattern (see below)")

parser.add_option('-s', '--string', default="MSTRG", help="if a different prefix is used for geneIDs assigned by StringTie [default: %default]")

parser.add_option('-k', '--key', default="prepG", help="if clustering, what prefix to use for geneIDs assigned by this script [default: %default]")

parser.add_option('-v', action="store_true", help="enable verbose processing")

parser.add_option('--legend', default="legend.csv", help="if clustering, where to output the legend file mapping transcripts to assigned geneIDs [default: %default]")

(opts, args)=parser.parse_args()

samples = [] # List of tuples. If sample list, (first column, path). Else, (subdirectory name, path to gtf file in subdirectory)

if (os.path.isfile(opts.input)):

# gtfList = True

try:

fin = open(opts.input, 'r')

for line in fin:

if line[0] != '#':

lineLst = tuple(line.strip().split(None,2))

if (len(lineLst) != 2):

print "Error: line should have a sample ID and a file path:\n%s" % (line.strip())

exit(1)

if lineLst[0] in samples:

print "Error: non-unique sample ID (%s)" % (lineLst[0])

exit(1)

if not os.path.isfile(lineLst[1]):

print "Error: GTF file not found (%s)" % (lineLst[1])

exit(1)

samples.append(lineLst)

except IOError:

print "Error: List of .gtf files, %s, doesn't exist" % (opts.input)

exit(1)

else:

# gtfList = False

## Check that opts.input directory exists

if not os.path.isdir(opts.input):

parser.print_help()

print " "

print "Error: sub-directory '%s' not found!" % (opts.input)

sys.exit(1)

#####

## Collect all samples file paths and if empty print help message and quit

#####

samples = [(i,glob.iglob(os.path.join(opts.input,i,"*.gtf")).next()) for i in next(os.walk(opts.input))[1] if re.search(opts.pattern,i)]

if len(samples) == 0:

parser.print_help()

print " "

print "Error: no GTF files found under base directory %s !" % (opts.input)

sys.exit(1)

RE_GENE_ID=re.compile('gene_id "([^"]+)"')

RE_GENE_NAME=re.compile('gene_name "([^"]+)"')

RE_TRANSCRIPT_ID=re.compile('transcript_id "([^"]+)"')

RE_COVERAGE=re.compile('cov "([\-\+\d\.]+)"')

RE_FPKM=re.compile('FPKM "([\-\+\d\.]+)"')

RE_TPM=re.compile('TPM "([\-\+\d\.]+)"')

RE_STRING=re.compile(re.escape(opts.string))

RE_GFILE=re.compile('\-G\s*(\S+)') #assume filepath without spaces..

#####

## Sort the sample names by the sample ID

#####

samples.sort()

#if opts.v:

# print "Sample GTFs found:"

# for s in samples:

# print s[1]

#####

## Checks whether a given row is a transcript

## other options: ex. exon, transcript, mRNA, 5'UTR

#####

def is_transcript(x):

return len(x)>2 and x[2]=="transcript"

def getGeneID(s, ctg, tid):

r=RE_GENE_ID.search(s)

#if r: return r.group(1)

rn=RE_GENE_NAME.search(s)

#if rn: return ctg+'|'+rn.group(1)

if r:

if rn:

return r.group(1)+'|'+rn.group(1)

else:

return r.group(1)

return tid

def getCov(s):

r=RE_COVERAGE.search(s)

if r:

v=float(r.group(1))

if v<0.0: v=0.0

return v

return 0.0

def getFPKM(s):

r=RE_FPKM.search(s)

if r:

v=float(r.group(1))

if v<0.0: v=0.0

return v

return 0.0

def getTPM(s):

r=RE_TPM.search(s)

if r:

v=float(r.group(1))

if v<0.0: v=0.0

return v

return 0.0

def is_overlap(x,y): #NEEDS TO BE INTS!

return x[0]<=y[1] and y[0]<=x[1]

def t_overlap(t1, t2): #from badGenes: chromosome, strand, cluster, start, end, (e1start, e1end)...

if t1[0] != t2[0] or t1[1] != t2[1] or t1[5]<t2[4]: return False

for i in range(6, len(t1)):

for j in range(6, len(t2)):

if is_overlap(t1[i], t2[j]): return True

return False

## Average Readlength

read_len=opts.length

## Variables/Matrices to store t/g_counts

t_count_matrix, g_count_matrix=[],[]

##Get ready for clustering, stuff is once for all samples##

geneIDs={} #key=transcript, value=cluster/gene_id

## For each of the sorted sample paths

for s in samples:

badGenes=[] #list of bad genes (just ones that aren't MSTRG)

try:

## opts.input = parent directory of sample subdirectories

## s = sample currently iterating through

## os.path.join(opts.input,s,"*.gtf") path to current sample's GTF

## split = list of lists: [[chromosome, ...],...]

#with open(glob.iglob(os.path.join(opts.input,s,"*.gtf")).next()) as f:

# split=[l.split('\t') for l in f.readlines()]

# if not gtfList:

# f = open(glob.iglob(os.path.join(opts.input,s[1],"*.gtf")).next())

# else:

# f = open(s[1])

with open(s[1]) as f:

split=[l.split('\t') for l in f.readlines()]

## i = numLine; v = corresponding i-th GTF row

for i,v in enumerate(split):

if is_transcript(v):

t_id=RE_TRANSCRIPT_ID.search(v[8]).group(1)

try:

g_id=getGeneID(v[8], v[0], t_id)

except:

print "Problem parsing file %s at line:\n:%s\n" % (s[1], v)

sys.exit(1)

geneIDs.setdefault(t_id, g_id)

if not RE_STRING.match(g_id):

badGenes.append([v[0],v[6], t_id, g_id, min(int(v[3]),int(v[4])), max(int(v[3]),int(v[4]))]) #chromosome, strand, cluster/transcript id, start, end

j=i+1

while j<len(split) and split[j][2]=="exon":

badGenes[len(badGenes)-1].append((min(int(split[j][3]), int(split[j][4])), max(int(split[j][3]), int(split[j][4]))))

j+=1

except StopIteration:

warnings.warn("Didn't get a GTF in that directory. Looking in another...")

else: #we found the "bad" genes!

break

##THE CLUSTERING BEGINS!##

if opts.cluster and len(badGenes)>0:

clusters=[] #lists of lists (could be sets) or something of transcripts

badGenes.sort(key=itemgetter(3)) #sort by start coord...?

i=0

while i<len(badGenes): #rather un-pythonic

temp_cluster=[badGenes[i]]

k=0

while k<len(temp_cluster):

j=i+1

while j<len(badGenes):

if t_overlap(temp_cluster[k], badGenes[j]):

temp_cluster.append(badGenes[j])

del badGenes[j]

else:

j+=1

k+=1

if len(temp_cluster)>1:

clusters.append([t[2] for t in temp_cluster])

i+=1

print len(clusters)

for c in clusters:

c.sort()

clusters.sort(key=itemgetter(0))

legend=[]

for u,c in enumerate(clusters):

my_ID=opts.key+str((u+1))

legend.append(list(itertools.chain.from_iterable([[my_ID],c]))) #my_ID, clustered transcript IDs

for t in c:

geneIDs[t]=my_ID

## geneIDs[t]="|".join(c) #duct-tape transcript IDs together, disregarding ref_gene_names and things like that

with open(opts.legend, 'w') as l_file:

my_writer=csv.writer(l_file)

my_writer.writerows(legend)

geneDict={} #key=gene/cluster, value=dictionary with key=sample, value=summed counts

t_dict={}

guidesFile='' # file given with -G for the 1st sample

for q, s in enumerate(samples):

if opts.v:

print ">processing sample %s from file %s" % s

lno=0

try:

#with open(glob.iglob(os.path.join(opts.input,s,"*.gtf")).next()) as f: #grabs first .gtf file it finds inside the sample subdirectory

# if not gtfList:

# f = open(glob.iglob(os.path.join(opts.input,s[1],"*.gtf")).next())

# else:

f = open(s[1])

transcript_len=0

for l in f:

lno+=1

if l.startswith('#'):

if lno==1:

ei=l.find('-e')

if ei<0:

print "Error: sample file %s was not generated with -e option!" % ( s[1] )

sys.exit(1)

gf=RE_GFILE.search(l)

if gf:

gfile=gf.group(1)

if guidesFile:

if gfile != guidesFile:

print "Warning: sample file %s generated with a different -G file (%s) than the first sample (%s)" % ( s[1], gfile, guidesFile )

else:

guidesFile=gfile

else:

print "Error: sample %s was not processed with -G option!" % ( s[1] )

sys.exit(1)

continue

v=l.split('\t')

if v[2]=="transcript":

if transcript_len>0:

## transcriptList.append((g_id, t_id, int(ceil(coverage*transcript_len/read_len))))

t_dict.setdefault(t_id, {})

t_dict[t_id].setdefault(s[0], fpkm)

t_id=RE_TRANSCRIPT_ID.search(v[len(v)-1]).group(1)

#g_id=RE_GENE_ID.search(v[len(v)-1]).group(1)

g_id=getGeneID(v[8], v[0], t_id)

#coverage=float(RE_COVERAGE.search(v[len(v)-1]).group(1))

#coverage=getCov(v[8])

fpkm=getFPKM(v[8])

transcript_len=0

if v[2]=="exon":

transcript_len+=int(v[4])-int(v[3])+1 #because end coordinates are inclusive in GTF

## transcriptList.append((g_id, t_id, int(ceil(coverage*transcript_len/read_len))))

t_dict.setdefault(t_id, {})

t_dict[t_id].setdefault(s[0], fpkm)

except StopIteration:

# if not gtfList:

# warnings.warn("No GTF file found in " + os.path.join(opts.input,s[1]))

# else:

warnings.warn("No GTF file found in " + s[1])

## transcriptList.sort(key=lambda bla: bla[1]) #gene_id

for i,v in t_dict.iteritems():

## print i,v

try:

geneDict.setdefault(geneIDs[i],{}) #gene_id

geneDict[geneIDs[i]].setdefault(s[0],0)

geneDict[geneIDs[i]][s[0]]+=v[s[0]]

except KeyError:

print "Error: could not locate transcript %s entry for sample %s" % ( i, s[0] )

raise

if opts.v:

print "..writing %s " % ( opts.t )

with open(opts.t, 'w') as csvfile:

my_writer = csv.DictWriter(csvfile, fieldnames = ["transcript_id"] + [x for x,y in samples])

my_writer.writerow(dict((fn,fn) for fn in my_writer.fieldnames))

for i in t_dict:

t_dict[i]["transcript_id"] = i

my_writer.writerow(t_dict[i])

if opts.v:

print "..writing %s " % ( opts.g )

with open(opts.g, 'w') as csvfile:

my_writer = csv.DictWriter(csvfile, fieldnames = ["gene_id"] + [x for x,y in samples])

## my_writer.writerow([""]+samples)

## my_writer.writerows(geneDict)

my_writer.writerow(dict((fn,fn) for fn in my_writer.fieldnames))

for i in geneDict:

geneDict[i]["gene_id"] = i #add gene_id to row

my_writer.writerow(geneDict[i])

if opts.v:

print "All done."
